# Interaction between the breast tumor microenvironment and gut microbiome

**DOI:** 10.1080/19490976.2025.2514136

**Published:** 2025-06-08

**Authors:** Grace Tang, Ewan K. A. Millar, Peter H. Graham, Julia Beretov

**Affiliations:** a Department of Medical Oncology, St. George Hospital, Kogarah, Australia; b St. George and Sutherland Clinical School, UNSW Sydney, Sydney, Australia; c Department of Anatomical Pathology, NSW Health Pathology, St. George Hospital, Kogarah, Australia; d Faculty of Medicine and Health Sciences, Sydney Western University, Campbelltown, Australia; e Cancer Care Centre, St George Hospital, Sydney, Australia

**Keywords:** Breast cancer, dysbiosis, microbiome, microbiota, estrabolome

## Abstract

Previously believed to be sterile, the breast microenvironment has been revealed by modern DNA sequencing technologies to harbor a diverse community of microorganisms. The breast tumor microenvironment (TME) has a microbial signature unique to that of other breast pathologies as well as between breast cancer subtypes and stage. Among the plethora of microorganisms identified, *Methylobacterium radiotolerans* and *Sphingomonas yanoikuyae* stand out, both elevated in breast cancer tissue and associated with cancer stage. Breast cancer is the most common malignancy affecting women and the second most common cause of cancer-specific death in women worldwide. Gut dysbiosis has recently emerged as a key player, although the exact mechanisms are still unclear. Hypothesized mechanisms include bacterial metabolites inducing genomic instability, imbalances in the local and systemic immune system, the role of gut microbiota in the regulation of estrogen metabolism. Probiotic commensals *Akkermansia muciniphila* and *Bifidobacterium* appear to have a protective effect, with evidence of gut wall protection, correlation with less advanced disease and better treatment efficacy and tolerability. This review outlines the relationship between the breast microbiome, the gut microbiome, the ‘estrabolome’, and the immune system in breast cancer. This characterization could make a significant clinical contribution, potentially leading to new methods of primary prevention, better prognostication and prediction, as well as new avenues of treatment.

## GRAPHICAL ABSTRACT

**Figure:**
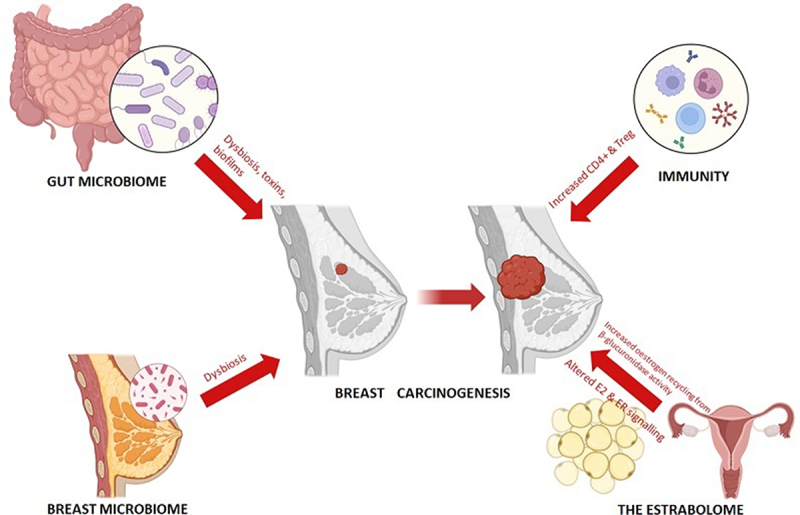
Created with BioRender.com

## Introduction

The breast microbiome is an emerging area of research that explores the presence, diversity, and potential influence of microbial communities within breast tissue. Interest in the human microbiome and its association with disease has seen a sudden increase in the number of publications (>12,000 in 2024 alone). With more than 15% of cancers globally now considered to be attributable to infectious causes,^1^ establishing the pathogenesis by which dysbiosis propagates tumorigenesis is of increasing relevance. The hype that currently surrounds the trillions of microorganisms (the microbiota) which call our bodies “home” as well as their genetic composition (the microbiome) needs to be carefully considered in light of the available evidence.^2^ Unlike the well-studied gut microbiome, the breast microbiome is less understood but is gaining attention for its potential role in breast health and disease. Molecular subtyping of breast cancer has critical prognostic and predictive value, routinely assessed by immunohistochemistry for hormone receptor and HER2 status. Estrogen receptor positive breast cancers have the greatest microbial diversity, while triple negative tumors have the least diverse TME.^3^

Breast cancer has a complex dynamic relationship between the breast microbiome, the gut microbiome, which includes estrogen metabolizing gut bacteria (the ‘estrabolome’) and the immune system. Breast cancer patients can be subdivided by their gut microbiome into four microbial clusters each with distinct gene expression, clinical characteristics and prognosis.^4^ The unfavorable clusters had high tumor mutational burdens and complex immune environments and interestingly included all molecular subtypes, albeit with a predominance of triple negative breast cancers, which typically have the worst clinical outcomes. Treatment response and outcomes are varied even within the same subgroup, generating interest in novel ways to categorize patients beyond the traditional system for a more tailored approach to treatment.

Gut dysbiosis with loss of diversity has been described in connection with many cancers, including breast, colorectal and lung cancer,^5–7^ and chronic diseases, with notable bacterial populations *Bacteroides*, *Prevotella* and *Ruminococcus* in chronic disease states.^8^ There is growing evidence that cancer cells may also be influenced by the pro-carcinogenic effects of microbial dysbiosis, via angiogenesis and increased T-helper 17 (Th17) response.^9,10^

Proving causality between the gut microbiome and the breast TME is a key topic to be explored in future research for the therapeutic potential of manipulating the microbiome is yet to be unlocked. Doing so could lead to major innovations in the approaches to managing breast cancer, shedding light on the gut microbiome as a prognostic biomarker or even target for precision medicine. In this review, we will outline the key evidence and potential mechanistic features which link the gut microbiome and the breast TME.

## Breast cancer

According to data from the Global Cancer Observatory, almost 2.3 million new cases and 700 thousand deaths from breast cancer were estimated in 2022.^11^ The 5-year overall survival for breast cancer is close to 90% in many developed countries, including the United Kingdom, America and Australia, but as low as 20% for metastatic disease.^12,13^

Breast cancer has four molecular subtypes: Luminal A, Luminal B, HER2 enriched and basal/triple negative. Whilst this classification is based on gene expression profiling, these equate to a simplified immunohistochemical tumor phenotype dependent on the expression of estrogen receptor (ER), progesterone receptor (PR), human epidermal growth factor receptor 2 (HER2) and proliferation marker Antigen Kiel 67 (Ki67) (Table 1). These features influence tumor behavior, guide treatment decisions and affect prognosis.^14^ However, this is further influenced by the complex TME of breast cancer, whereby single cell transcriptomics investigations on the role of tumor infiltrating lymphocytes (TILs) and stromal cells in disease progression and treatment response have been identified.^16^

**Table 1.** Breast cancer subtypes.^14,15^

Cancer subtype	Biomarker			Prevalence (%)	Systemic Treatment	Characteristics	Prognosis
	ER	PR	HER2/Ki67				
Luminal A	+	±	-/low	40–50	Hormone therapy e.g. SERM, ER antagonist, AI Ovarian ablation e.g. oophorectomy, LHRH analogues, CDK inhibitors	Slow growth Less aggressive Better hormonal therapy response	Best
Luminal B	+	±	+/high	10–20		Higher proliferation than luminal A	Worse than luminal A
HER2	–	–	+/any	10	Anti-HER2 therapies Tyrosine kinase inhibitor mTOR inhibitor (everolimus)	Aggressive	Poor short term
TNBC/Basal-like	–	–	–/any	10–15	None; chemotherapy	Aggressive Found in African Americans, premenopausal & younger women BRCA1 mutation associated	Poor short-term

AI = aromatase inhibitor; CDK = cyclin-dependent kinase; ER= estrogen receptor; LHRH = luteinizing hormone releasing hormone; mTOR = mammalian target of rapamycin; PR= progesterone receptor SERM = selective ER modulator; TNBC = triple negative breast cancer.

Importantly, the risk of developing breast cancer rises with increased circulating estrogen levels, brought on by metabolism of endogenous hormones and exogenous gut estrogens.^17^ Linked with increased risk is exposure to xenobiotics (drugs, chemicals, and environmental pollutants) and their impact on the gastrointestinal and mammary microbiota.^18^

## Breast microbiome

The human breast microbiome is unique and distinct from the microbial composition of other organs,^19^ although an unequivocal profile has yet to be established. Investigation of the breast microbiome has documented a diverse population of bacteria in breast tumors that differs significantly from normal breast tissue, irrespective of age, geographical location, obstetric history and cancer-status. *Proteobacteria, Actinobacteria* and *Firmicutes* are the dominant phyla identified, with samples studied including The Cancer Genome Atlas (TCGA) breast cancer data study by Thompson et al.^20^ and the large tissue sample study by Tzeng et al.^20–26^ A profile of the human mammary microbiome using 16S rRNA sequencing and culture also reported *Proteobacteria* as the most common phylum, followed by *Firmicutes, Actinobacteria* and *Bacteroidetes* independent of lactation history.^25^

The composition of the breast microbiome at the phylum level has been validated in multiple subsequent studies. Nejman et al.^27^ published a landmark publication profiling the microbiome of over 1,500 tumor and normal tissue samples, highlighting the diverse microbiome of breast cancer tissue. Notably, >60% of breast cancer samples were positive for bacterial DNA with an average of 16.4 species identified in each.^27^ Bacterial species of note include *Escherichia coli, Sphingobacteria, Prevotella*, *Mobiluncus*, *Brevundimonas Staphylococccus Methylobacterium radiotolerans* and *Sphingomonas yanoikuyae*. Additionally, *Bacteriodes*^20,22,23,25,26^ and *Bifidobacteria* (from phyla *Bacteroidetes* and *Actinobacteria*, respectively) were found as dominant genera.^28^

The breast microbiome may influence tumor behavior through several mechanisms: inflammation, immune modulation, metabolism and hormonal regulation. Still unknown is whether it is local dysbiosis that promotes carcinogenesis or whether the tumor microbial signature is naturally selected by the TME. Current findings highlight unrecognized links between breast dysbiosis and breast cancer. A summary of the major findings in the breast cancer microbiome are detailed in Table 2.

**Table 2.** Summary of abundant bacterial species associated with the breast microbiome.

Reference Chronological order	Study Size	Breast Sample Type	Method of Analysis	Abundant Bacteria in Breast Cancer Tissue
Urbaniak et al. ^25^	60 BC samples **Canadian study**: 27 BC & adjacent 11 Benign disease 5 healthy Ctls **Irish study**: 33 BC & adjacent 5 healthy Ctls	Frozen	16S rRNA gene seq Bacterial culture	Proteobacteria: *E. coli* **Canadian BC samples**: *Acinetobacter* (10%), *Bacillus* (11.4%), *Enterobacteriaceae* (8.3%) **Irish BC samples**: Proteobacteria *Enterobacteriaceae* (30.8%), *Staphylococcus* (12.7%), Listeria *welchimeri* (12.1%)
Xuan et al.^6^	Analysis 1: 20 ER+ tumor 20 adj normal Analysis 2: 39 tumor 39 adj normal 23 healthy controls	FFPE	16S DNA pyro-seqencing qPCR	**Abundant phyla in all samples**: *Proteobacteria, Firmicutes, Actinobacteria, Bacteroidetes* Pseudomonas: M. *radiotolerans* Adjacent tissue: *S. yanoikuyae*
Banerjee et al.^29^	100 TNBC 37 **Controls**: (17 matched, 20 non-matched)	FFPE	PathoChip microarray PCR MiSeq	*Arcanobacterium* (75%), then *Brevundimonas, Sphingobacteria, Providencia, Prevotella, Brucella, Eschherichia, Actinomyces, Mobiluncus* & *Propionibacteria, Geobacillus, Rothia, Peptinophilus, Capnocytophaga*
Chan et al.^30^	25 ductal carcinoma 23 healthy ctls	NAF	16S rRNA gene amplicon sequencing	**NAF**: Firmicutes, Proteobacteria and Bacteroidetes ***NAF** (BC history): Alistipes (phylum Bacteroidetes)* **Controls**: *Sphingomonadaceae*
Hieken et al.^23^	15 invasice BC samples, 13 benign disease no atypia. 28 adj ctls	Frozen Adjacent normal Adjacent skin	16S rRNA sequencing	Fusobacteriota: Fusobacterium *Atopobium, Gluconacetobacter, Hydrogenophaga, Lactobacillus*
Urbaniak et al.^26^	45 BC + normal adj 13 Benign breast disease 23 healthy ctls	Frozen	16S rRNA amplicon sequencing	Predominant: Firmicutes (phlya Bacillus & Lactococcus), Proteobacteria, Actinobacteria, Bacteroidetes (Prevotella) **BC**: genus *Bacillus, Enterobacteriaceae, Staphylococcus, Comamondaceae, Bacteroidetes*. **Healthy control**: *Prevotella, Lactococcus, Streptococcus, Corynebacterium*
Yazdi et al.^31^	123 sentinel BC-LN + adj normal 5 healthy breast	Frozen Frozen LN	RT-PCR	*M. radiotolerans* increases in sentinel LN with increasing BC stage. **Normal breast tissue**: Proteobacteria- Sphingomonas *yanoikuyae*
Thompson et al.^20^	688 tumor tissues 72 adjacent normal	The Cancer Genome Atlas (TCGA) BC data	16S rRNA gene seq	*Proteobacteria, Mycobacterium fortuitum* & *phlei*. **Adjacent normal**: *Actinobacteria*
Wang et al.^32^	57 invasive BC + adj normal 21 healthy ctls	Frozen	16S rRNA gene sequencing	**BC HR+**: lower abundance of Proteobacteria: Methylobacterium compared to control, increased *Alcaligenaceae* (unidentified genus)
Banerjee et al.^21^	148 BC (50 ER+/PR+, 34 hER2+, 24 triple positive, 40 TN) 20 healthy ctls: non-matched breast tissues	FFPE	Whole genome & transcriptome amplification PathoChip microarray PCR Sanger seq	**All four BC types**: *Proteobacteria* (genera Brevundimonas, Bartonella), *Firmicutes* & *Actinomyces (sp. Mobiluncus)*. **ER/PR positive**: *Arcanobacterium, Escherichia Bifidobacterium, Cardiobacterium, Citrobacter*, **HER2 positive**: *Streptococcus* **Triple positive**: *Bordatella, Campylobacter, Chlamydia, Chlamydophila, Leionela, Pasteurella* **TNBC** least microbially complex; associated with *Aerococcus, Arcobacter, Geobacillus, Orientia, Rothia*. Prevotella only in BC samples.
Costantini et al.^22^	16 BC patients (12 core biopsies, 7 surgical excision) 19 adjacent breast healthy ctls	Fresh	Microbial gDNA isolation 16S rRNA gene amplicon sequencing	*Proteobacteria, Firmicutes, Actinobacteria, Bacteroidetes; Ralstonia* (most abundant genus): *Methylobacterium* and *Ralstonia* (phylum Proteobacteria) relatively increased, *Sphingomonas* decreased
Meng et al.^28^	72 IDC 47 ER+; 25 ER- 42 PR+; 30 PR- 22 benign ctls:	Frozen	16S rRNA gene amplicon seq	Actinobacteria: Agrococcus, Propionicimonas *families* Micrococcaceae *Caulobacteraceae, Rhodobacteraceae, Nocardioidaceae, Methylobacteriaceae* **Increasing BC grade**: Agrococcus *abundance &* reduction in *Bacteroidaceae*
Chiba et al.^33^	15 IDC (previous neoadjuv chemo) Ctls: 18 IDC no previous chemo	Frozen tissue Cell lines: MDA-MB-231 MB-4T1 ZR-75–1, 67NR MCF-7	PCR, IHC 16S rRNA seq Microarray staining Western blot Proliferation assay	**IDC Neoadjuvant chemotherapy**: *Pseudomonas*, decrease Prevotella. *P. aeruginosa* stimulates cell growth: MDA-MB-231, MBF-7 & ZR-75–1 **Metastasised tumors**: *Brevundimonas* & *Staphylococcus*
Nejman et al.^27^	355 BC samples 270 ER+; 49 ER- 61 hER2+ 247 hER2- 36 TN; 284 non-TN 256 adj normal	Frozen FFPE	16S real-time qPCR 16S rRNA gene amplification CLEM IHC, FISH, IF	In descending order: *Streptococcus infantis, Lactobacillus iners, Corynebacterium and Fusobacterium nucleatum, Staphylococcus cohnii, Paracoccus marcusii, Enterobacter cloacae, Acinetobacter, Staphylococcus aureus*
Thyagarajan et al.^34^	23 BC samples	Frozen	16S rRNA amplicon sequencing MiSeq	In descending order: *Ralstonia, Staphylococcus*, family *Bradyrhizobiaceae, Rubrobacter, Pseudomonas*
Tzeng et al.^24^	221 BC samples 87 healthy ctls	Frozen	16S rRNA gene seq 16S qPCR IHC NanoString	*Pseudomonas, Proteus, Porphyromonas* & *Azomonas* **Increased with higher stage**: *Porphyromonas, Lacibacter, Ezakiella* and *Fusobacterium*. **Benign breast tissue**: genera *Propionibacterium, Finegoldia, Granulicatella, Streptococcus, Anaerococcus, Ruminococcaceae UCG-002, Corynebacterium 1, Alicyclobacillus, Odoribacter, Lactococcus, Esherichica/Shigella*

Adj: adjacent; Chemo: chemotherapy; CLEM: correlative light and electron microscopy; Ctls: controls; FFPE: formalin fixed paraffin embedded; ER+: estrogen receptor positive; FISH: fluorescence in situ hybridization; HER2+: human epidermal growth factor receptor 2; HR: hormone receptor; IDC: invasive ductal carcinoma; IF: immunofluorescence; IHC: immunohistochemistry; LN: lymph nodes; NAF: nipple aspirate fluid; OTU: operational taxonomic unit (sequence identity-based clustering); PCR: polymerase chain reaction; PR+: progesterone receptor positive; Sequencing: seq; TNBC: triple negative breast cancer.

### Bacterial species associated with breast cancer

#### Escherichia coli

*Escherichia coli* is found abundant in normal breast tissue adjacent to breast tumors in comparison with healthy controls.^25,26^ *E. coli* has various virulence factors that promote adhesion, infiltration and survival, as well as a genotoxic effect, including the polyketide synthase (*pks*) gene.^35^ Strains of *E. coli* with the *pks* pathogenicity island are able to produce colibactin, which can induce DNA damage, contributing to gene instability and carcinogenesis.^26^

#### Sphingobacteria, Prevotella (both phylum Bacteroidetes)

A relative abundance of *Sphingobacteria* and *Prevotella* has been detected in triple negative breast cancer (TNBC) samples;^29^ others report reduced numbers in breast cancer tissue relative to healthy control breast tissue, receptor status unspecified,^6,26,30^

#### Actinomyces (family Actinomycetaceae), family Propionobacteraceae (both phylum Actinobacteria)

Also elevated in breast cancer tissue are genera *Actinomyces*, as well as family *Propionobacteraceae* (both from phylum *Actinobacterium*) along with *Staphylococcus*,^21,26,29^ all gram positive bacteria, which can induce interferon-gamma (IFN-γ) secretion from T -cells and natural killer (NK) cells.^36^ Traditionally IFN-γ is known to be cytotoxic and inhibitory of tumors,^36^ with evidence that IFN-γ is lost early in breast cancer.^37^ IFN-γ is now thought to be able to promote tumor growth and survival in certain environments via immunosuppression, immuno-resistance and upregulation of cell proliferation.^38^

#### Staphylococcus and Brevundimonas

Overall, *Staphylococcus* was found to be more abundant in breast cancer tissue and found by Chiba et al.^33^ to be more abundant in metastatic disease along with a higher abundance of *Brevundimonas*.^21,25–27,29,33^ *Brevundimonas* concentrations were found to be significantly increased in tumors of all breast cancer subtypes.^21^

Notably, strains of *E. coli* and *S. epidermidis* demonstrated the ability to cause DNA damage by inducing double-stranded DNA breaks in HeLa cells.^26^

#### Methylobacterium radiotolerans

*Methylobacterium*, family *Methylobacteriaceae*, is reported to be elevated in breast cancer tissue^6,22,28^ and the sentinel lymph nodes of breast cancer patients, and associated with increasing cancer stage.^31^ Intratumoral *Methylobacterium* has been significantly associated with worse prognosis in gastric cancer, with a causal role via reducing CD8+ T cell response at the tumor site.^39^ In prostate cancer, *M. radiotolerans* is overrepresented in the tumor microbiome, and negatively associated with staging.^40^

Contrary to these findings, Wang et al., reported the depletion of *Methylobacterium* in hormone receptor (HR) positive breast cancer tissue.^32^ This disparity may be in part due to differences in methodology (e.g. formalin-fixed paraffin embedded vs. fresh vs. frozen tissue, choice of DNA extraction kit). Additionally, heightened invasive potential in breast cancer has been correlated to tumors with a reduced abundance of *Methylobacterium*.^2^ *Methylobacteriaceae*, produces phytohormones, which have an anti-cancer effect by inhibiting proliferation and inducing apoptosis, demonstrated in breast cancer cells *in vitro*.^41,42^

### Protective bacteria abundant in non-cancerous breast tissue

The breast microbiome may play a crucial role in maintaining breast health, influencing local immune responses and potentially protecting against pathogens. Several studies suggest that a balanced microbiome may support anti-inflammatory and immunomodulatory functions, contributing to tissue homeostasis and defense mechanisms. Prominent bacteria isolated from benign breast tissue, including non-cancerous adjacent breast tissue, compared to cancerous breast tissue include: *Corynebacterium, Lactococcus* and *Streptococcus*;^24,26^ *Prevotella*;^26^ *Methylobacterium*;^32^ *Sphingomonas*;^6^ *Escherichia*^20,24^ and *Haemophilus*.^20^ *Sphingomonadaceae* was found more abundant in the nipple aspirate fluid of women without a history of breast cancer compared to that taken from women with a prior history of breast cancer.^30^ Additionally, *Alicyclobacillus, Anaerococcus, Finegoldia, Granulicatella, Odoribacter, Propionibacterium, Ruminococcaceae* and *Shigella* have been reported to be relatively abundant in healthy breast tissue.^24^

*Lactococcus*, a genus of lactic acid bacteria, has been shown to be protective against breast cancer by altering the cytotoxic effect of NK cells, which enhance cellular immunity and inhibit tumor growth, demonstrated in both tissue from humans with solid organ tumors and mice breast cancer models.^43–45^ In mice, oral administration of *Lactobacillus* was shown to suppress breast cancer growth and metastasis.^46^

*Propionibacterium* and *Streptococcus* are associated with T cell activation genes.^24^ *Propionibacterium*, which has anti-tumoral properties, is relatively depleted in breast cancer tissue.^24^ *Propionibacterium* induces an anti-cancer effect via activation of NK cells, macrophages, and T cells.^47^ This genus has also been associated with lower levels of oncogenic growth factors, and its downregulation is speculated to promote tumor growth through inhibition of the adaptive immune response.^24^ In vivo breast tumor suppressing efficacy has been demonstrated by inhibiting peritumoral angiogenesis and promoting apoptosis.^47^ *Streptococcus* has an anti-oxidant effect and is able to neutralize peroxides and reactive oxygen species, protecting cells against DNA damage.^48^ In addition, *Streptococcus* has been studied for its anti-tumor effect via promotion of Toll-like receptor (TLR) 4 signaling.^49,50^ It also produces cadaverine, which inhibits breast cancer invasion and epithelial-to-mesenchymal transition.^51,52^

*Odoribacter* (order *Bacteroidales*), found relatively abundant in benign breast tissue samples,^24^ produces the short chain fatty acid butyrate, which has been shown to have anti-cancer properties through its anti-inflammatory activity.^52^

Absolute abundance of *S. yanoikuyae* is increased in adjacent non-cancerous tissue.^6,22^ *Sphingomonadaceae* (particularly genera *Sphingomonas, Sphingobium, Novosphingobium*, and *Sphingopyxis*) were shown to degrade aromatic hydrocarbons such as estrogens, which is particularly relevant in HR positive breast cancer.^53–56^ *Sphingomonas* is found in breast tumors with increasing breast cancer stage^31^ with evidence of a protective effect demonstrated in ER positive breast cancers.^57^ Importantly, *S. yanoikuyae* expresses glycosphingolipid ligands that activate invariant NK cells,^58^ which are mediators of tumor immunosurveillance^59^ and breast cancer metastasis.^60^

## Immunological profile of the breast TME

Breast tumorigenesis attracts a significant influx of immune cells, both adaptive and innate, to the breast TME. The immune profile of the tumor microbiome modulates the local inflammatory response that precedes tumor formation and contributes to progression.^61^ Disequilibrium between inflammatory and immunosuppressive signals from the immune system is a key player in the development and progression of carcinogenesis.^62^ Breast tumors demonstrate an influx of immunosuppressive type cells over proinflammatory immune cells.^63^

Previously thought to have low lymphocyte infiltration and to be immunologically “cold”, breast tumors have recently been found to demonstrate immunological heterogeneity between patients and tumor types.^64^ Tumor infiltrating lymphocytes (TILs) have an anti-tumoral effect, given their various cytotoxic and cytokine inducing abilities, as well as a role in recognition of tumor antigens. T-helper 1 (Th1) lymphocytes facilitate tumor suppression and upregulation of inflammatory cytokines, such as IFN-y, TNF-α and IL-12, to facilitate tumor cell apoptosis and promote cytotoxic CD8+ T cell activity.^65–67^ Permeation of TILs by CD8+ T cells is positively correlated with survival and therapeutic efficacy.^68,69^ Conversely, T-helper 2 (Th2) CD4+ lymphocytes have been associated with pro-tumorigenesis via IL-4 and IL-13 mediated promotion of mammary tumor cell invasion and metastasis.^70,71^ Other mechanisms described include resting CD4+ T cells being converted into regulatory T (Treg) cells, which support immune escape and have been shown to promote metastasis of breast cancer cells;^72^ whereas CD8+ T cells inhibit breast tumors via suppression of RANK signaling.^73^

Treg cells are found in higher numbers in invasive breast cancers than in-situ breast cancers, in ER negative more so than ER positive breast tumors and are associated with higher rates of relapse and poorer survival.^74,75^ Treg cell infiltration may be useful as a prognostic marker for identifying patients with a favorable benefit-to-risk ratio for undergoing further hormonal therapy beyond the standard dose. Estradiol has been found to promote proliferation of Treg cells^76^ so it follows that inhibition of estrogen in the TME may also inhibit Treg cell numbers. Treatment of breast cancer patients with the aromatase inhibitor letrozole causes a significant reduction in Treg cells in the TME (*p* < 0.0001)^77^ and in ER positive breast cancer patients increases tumor infiltration by B cells and CD4+ T cells.^78^

TNBC has a poor prognosis with no targeted therapies and few systemic therapeutic options other than chemotherapy.^79^ High peri-tumoral stromal TIL density is most often seen in TNBC and HER2 positive breast cancers, described to comprise mainly of T cells CD4+ and CD8+ and, CD19+ B cells and NK cells, along with increased PD-L1 expression.^80,81^ It is associated with improved prognosis and is a positive predictive factor for the efficacy of neoadjuvant chemotherapy.^82–84^ There is also interest in TIL expression in residual tumor post-neoadjuvant therapy given the growing role of immunotherapy in breast cancer treatment.^85^ Targeted immunotherapy with programmed death-ligand 1 (PD-L1) or programmed cell death protein 1 (PD-1) monoclonal antibodies is now offered to patients with early and metastatic TNBC, yielding promising results.^80,86^

## Next generation sequencing technology

### Gut microbiome 16S gene sequencing vs. Shotgun metagenomics

Current molecular techniques employed to study the microbiome largely involve Next Generation Sequencing (NGS) – either 16S ribosomal RNA (rRNA) sequencing or shotgun metagenomics.^87^ While 16S rRNA gene sequencing is routine for profiling bacterial gene expression and is useful when sequencing large numbers of samples, it provides limited data on taxonomy to differentiate between species of bacteria.^88^ The accuracy and reproducibility of bacterial identification by 16S rRNA sequencing is affected by the sampling technique and DNA isolation kit used, particularly its efficacy at cell lysis.^89^ Recent advancements and novel gene sequencing have provided even more detail about pathogens, such as organism composition and specific immune response. An example is dual RNA sequencing (RNAseq), which allows a parallel transcriptomic analysis of bacterial pathogens and their eukaryotic host cells, and single cell RNAseq, which has enabled identification of cells containing bacteria, and provided information on the interactions between immune cells and microbes during infection.^90^

Shotgun metagenomics provides taxonomic data on all species present in a sample (i.e. bacteria, viruses, archaea, protozoa, fungi) and information on potential metabolic output through analysis of all genes present, creating a more complete picture of the microbiome.^91^ On the down side, metagenomics is more expensive and detection is confounded by contaminants during the preparation/DNA extraction phase from kits or laboratory agents.^91,92^

Direct comparison of the two methods on the same fresh frozen stool samples demonstrate shotgun metagenomics permits for a more extensive characterization of the microbiome and can identify more bacterial species even at low sequencing depths.^92^ Comparison of oral and gut microbiomes yielded significant community differences with 16S rRNA sequencing analysis at the genus and species level, while shotgun metagenomics demonstrated a larger proportion of shared taxonomy between oral and gut microbiomes with improved resolution at the species level.^93^

## Gut microbiome and the immune response

Microorganisms such as bacteria, archaea, viruses, protozoa and fungi can be found widely throughout the body’s epithelial surfaces. Bacteria from the gastrointestinal tract (GIT) can influence not only the local but also the systemic immune system via the enterohepatic circulation.^94,95^ It is by this mechanism that distant tumors can be exposed to microorganisms of the GIT.

Remarkably, with approximately 2 kg of microorganisms existing in the gut, the metabolic output of the GIT is equivalent to that of the liver.^96^ The GIT contains approximately 1000 species of commensal microorganisms distributed along its length,^97^ generally becoming more abundant distally – with 10^2^–10^3^ bacteria per mL in the stomach and up to 10^12^ per mL in the colon.^98^ Their aggregated bacterial genomes comprise 150 times more genes than their host human genome.^99^

The composition of the gut microbiome is highly variable between individuals and is influenced by factors such as diet, ethnicity, geographical location, drug history, body mass index (BMI), exercise and environmental interactions.^95,100^ The GIT is exposed to a plethora of external antigens that can trigger immune responses.^98^ The dominant gut microbes come from four major phyla: *Firmicutes, Bacteroidetes, Proteobacteria* and *Actinobacteria*, with approximately 90% of the microbiome represented by the genus *Faecalibaterium* of phylum *Firmicutes*, and genera *Bacteroides* and *Alistipes* of phylum *Bacteroidetes*.^98,101,102^ The *Actinobacteria* phylum is proportionally less abundant and mainly represented by the *Bifidobacterium* genus.^101^

**Figure 1.**
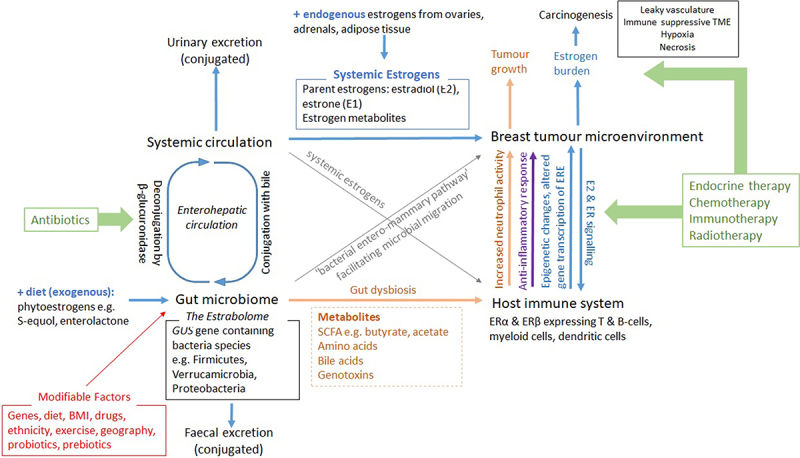
Mechanisms of bacterial and metabolite invasion of the colonic mucosa.

Commensal gut microbes colonize the GIT and are essential for metabolic and immune functions with regions of the GIT affected by specific bacterial density and diversity.^96,103^ *Clostridia* species and *Bacteroides fragilis*, which tend to be found in the colon, are shown to promote production of immune suppressor Treg cells.^104,105^ Despite innate mechanisms in place at the gut lumen to prevent penetration of gut bacteria, microbes and their metabolic products may still cross the epithelium into the systemic circulation (Figure 1). The breast and GIT are connected via the mucosal immune system, such that infection or immunization of one mucosal organ can induce immunoglobulin A (IgA) production at a distal mucosal site and protect it from the same infection.^108^ Characterizing the association between breast cancer and gastrointestinal dysbiosis would be of interest in establishing a relationship in pathogenesis.

Microorganisms on the luminal side of the gut epithelium are equipped with various mechanisms to penetrate the epithelium and migrate into the systemic circulation. Migration can occur via a paracellular pathway for small-diameter organisms, and transcellularly for larger organisms via transcytosis.^106^ Bacteria can have virulence factors such as: Lipopolysaccharides (LPS), an endotoxin found in gram-negative bacteria, which can activate toll-like receptor 4 (TLR4), CD14 and scavenger receptor-BI (SR-BI) to induce a pro-inflammatory response and allow its transcellular migration across the gut epithelium;^106,107^ strains of Escherichia coli containing the pks gene cluster and colibactin induces double-stranded DNA breaks and gene instability in host cells.

Some gut bacteria are protective and can produce short chain fatty acids, which have been shown to reduce inflammation and strengthen the intestinal epithelium by increasing production of Treg cells. Treg cells have anti-inflammatory properties and promote differentiation of effector T-helper cells. Proliferation of Treg cells suppresses effector T-cell immune responses, leading to reduced local inflammation. Described to have an immunosuppressive effect, *B.* *fragilis* contains polysaccharide A in its capsule, which through ligation with TLR2 mediates the differentiation of T reg cells to produce immunosuppressive IL-10, which in turn suppresses IL-17 production.

Once on the baso-lateral side of the gut epithelium, antigens can be recognized by dendritic cells in Peyer’s patches and mesenteric lymph nodes, which will trigger CD4+ T and B cells to respond. The subsequent systemic reaction means the lymphocytes, with their infection-specific receptors, can reach other organs of the mucosal immune system.^108^ Once arrived at effector sites, the plasma cells are transported to the apical side of the epithelial cells and release secretory IgA antibodies specific to antigens encountered at the induction site.^108^

Created with BioRender.com

### Diet

Diet has a modifiable role in shaping the composition of the gut microbiome, with most of this variation found to be in the ratio of *Prevotella* to *Bacteriodes*, both from the *Bacteroidetes* phylum. The two genera show an inverse relationship, with *Prevotella* found more in the gut of hosts with a non-Western diet consisting abundantly of plants, fiber and polysaccharides; whereas *Bacteroides* is found to dominate in hosts with a Western diet comprising more proteins and fats.^109^ More specifically, dietary fiber from fruits and vegetables is associated with colonization with *Clostridia*, while fiber from beans raises the prevalence of *Actinobacteria* and *Bifidobacteriales*.^110^ A plant-rich diet is associated with a lower incidence of colorectal cancer,^111^ due to its anti-inflammatory effect on Treg cell induction.^112^

### BMI

The association between breast cancer incidence and a higher BMI is well studied. Increased systemic estrogens is seen in obesity, from aromatization of abundant adipose tissue.^113^ This is particularly the case in post-menopausal women, where the aromatization of androgens and adipose tissue becomes the primary source of estrogens.^114^ In breast cancer, the percentage of body fat appears to be related to *Akkermansia muciniphila* abundance along with higher levels of *Prevotella*, *Lactobacillus*, and lower levels of *Clostridium*, *Campylobacter* and *Helicobacter*.^115^

### Short chain fatty acids

Intestinal microbiota can directly influence the host immune response via the production of metabolites, such as short chain fatty acids (SCFA),^116^ amino acids and vitamins.^117^ Obese and overweight patients have significantly higher levels of fecal SCFA, *Firmicutes* and a higher *Firmicutes* to *Bacteroidetes* ratio than normal-weight patients, independent of diet.^118^ The *Firmicutes* to *Bacteroidetes* ratio is a marker of gut dysbiosis and positively correlated with obesity and inflammation.^119^ Prominent gut SCFAs, propionate, butyrate and acetate have been shown to reduce inflammation and strengthen the intestinal epithelium.^120,121^ Major metabolite butyrate has local anti-neoplastic effects and promotes normal colonic growth and differentiation of mucosa.^122^ Butyrate inhibits the production of inflammatory cytokines, including TNFα, IL-6, IL-8.^123^ Another SCFA, cadaverine, is thought to be a pro-carcinogenic amine^124^ with some studies reporting elevated levels in cancer^125^ and others reporting decreased levels.^126^ However, in breast cancer, cadaverine appears to be a tumor suppressor, reported to cause lower tumor mass and invasion, less metastases and lower tumor grade.^127^ Gut commensals are also able to produce SCFA, including *Clostridia* and *Akkermansia* species.^115,128^ *Clostridium butyricum* has been shown to reduce lymphocyte counts, enhance immunity and improve the ratio of beneficial to pathogenic bacteria in gastric cancer patients.^128^ Importantly, *A. mucinophilia* thickens the gut mucosal layer, which impedes bacterial translocation.^115^

### Microbial toxins

The role of bacteria in cancer development and therapy has been well known for almost a century.^129^ Microbes are thought to promote carcinogenesis by induction of DNA damage via toxins, the most well known being *Helicobacter pylori* causing gastric cancer.^130^ Changes in the composition of the gut microbiome can cause inflammation and genotoxic stress via bacterial toxins, which damage the mucosal barrier allowing bacterial infiltration.^131^

Lipopolysaccharides (LPS), found in the cell wall of gram-negative bacteria, is a virulence factor that promotes inflammation through cytokine stimulation production by binding to TLR4.^132^ Involved in tumor survival and progression, TLR4 is upregulated in HR positive (MCF-7) and triple negative (MDA-MD-231) human breast cancer cell lines and is linked to increased metastasis.^133^ LPS has been reported to increase PD-L1 expression in gastric cancers, which when bound to the PD-1 transmembrane receptor inhibits T-cell activation.^134^ Via induction of the TLR4/NF-KB signaling pathway, LPS has been reported to promote cancer cell adhesion, invasion and metastasis.^135–139^

Similarly, strains of *Bacteroides fragilis*, which secrete the *B. fragilis* toxin (BFT), have been found to cause intestinal inflammation and promote colon tumor cell survival and proliferation. It degrades E-cadherin in the colon, leading to epithelial cell damage, inflammation and increased metastatic potential.^140^ The toxin can activate signal transducers and activators of transcription (STAT) 3 and T helper 17 (Th17) cell mediated responses leading to an upregulation of IL-17 levels, involved in intestinal inflammation.^140^ On the other hand, *B. fragilis* appears to have an opposite effect on the adaptive immune system. It has been described to have an immunosuppressive effect via Treg cells, as it contains polysaccharide A in its capsule, which through ligation with TLR2 mediates the differentiation of Treg cells to produce immunosuppressive IL-10, which in turn suppresses IL-17 production.^141,142^ *B. fragilis* has been identified in breast tissue, both benign and cancerous, and nipple aspirate fluid (NAF), and strains producing BFT have been demonstrated in mice models to cause local inflammation and epithelial hyperplasia.^143^ Additionally, mammary or gut colonization with BFT-bearing *B. fragilis* increased disease progression and metastases.

### Biofilms

Biofilms can form on surfaces in the body from a community of microorganisms that adhere to each other within an extracellular matrix. Recent studies investigating the role of bacterial biofilms in colon carcinogenesis found that colonic biofilms upregulate polyamine metabolites to promote proliferation and tumor growth.^144–146^ Gut biofilms, suggested to be drivers of gut carcinogenesis, contain bacteria that promote DNA damage in colonic epithelium leading to initiation of tumor formation, although the exact mechanisms and species are still unknown.^147–149^ A study investigating the presence of bacterial biofilms in malignant breast wounds reported a predominance of anaerobic bacteria, although biofilm formation was not found to be associated with a particular species of bacteria nor clinical signs.^150^ A symbiotic relationship may exist, whereby biofilms may provide a protective environment for cancer cells allowing them to evade host immune responses.^151^

Interestingly, it has been proposed that certain drugs used in cancer therapy, such as doxorubicin, promote the formation of biofilms *in vitro*,^152^ with the potential to inhibit cancer metastasis or even act as a vector for cancer therapies.^129,153^

### Microbial dysbiosis

Shotgun metagenomic studies exploring stool samples confirmed 38 bacterial species enriched in postmenopausal breast cancer patients and healthy controls, including *E. coli, Klebsiella, Prevotella amnii, Enterococcus gallinarum* and *Actinomycess*.^119,154^ Gut and mammary dysbiosis, resulting in an imbalance and loss of diversity in microbial composition, has been found to be associated not only with breast cancer development but with specific subtypes^2,21,117^ and responsiveness to therapy.^155,156^

The gut microbiome plays a critical role in modulating the host immune response. Gut dysbiosis may alter the balance between pro-inflammatory and anti-inflammatory immune responses, potentially impacting tumor surveillance and immune-mediated tumor suppression. The induction of inflammation even in non-cancerous mammary tissue suggests a possible pathway by which early gut dysbiosis is able to remotely contribute to breast carcinogenesis, as mammary inflammation has been linked to increased breast cancer risk.^157^ Mice transplanted with HR positive breast tumors demonstrated that pre-cancerous gut dysbiosis influences host immunity both systemically and locally, early dysbiosis enhances serum concentrations of cytokines (IL-22 and IL-23) and chemokines (GM-CSF, CCL2 and CXLC2), and increases circulation of cancer cells.^158^ Within the breast TME, gut dysbiosis promoted fibrosis and inflammation, with significantly upregulated CXCL10 and CCL2 chemokines, and myeloid infiltration both in the tumor and adjacent mammary gland.^158^

Shi et al.^81^ looked at TIL abundance in breast tumors and demonstrated a link between the gut microbiome and the TME. Higher abundance of TILs in breast tumors remains associated with a higher gut abundance of genera including *Mycobacterium, Rhodococcus*, and *Catenibacterium*, while low tumor abundance of TILs was associated with lower gut concentrations of *Methanosphaera* and *Anaerobiospirillum*.^81^ Also, a higher fecal abundance of *Barnesiae* (genus *Bacteroides*), involved in estrogen metabolism, is associated with low TIL abundance.^81^

Another pathway by which gut dysbiosis can lead to breast tumorigenesis may be via host immune responses to gastrointestinal infection. Infection of mice with *Helicobacter hepaticus*^159^ demonstrated increased levels of neutrophils in mammary tissue and amplified risk of breast carcinogenesis, while depletion of neutrophils inhibited tumor growth.^160,161^ Significant bacterial species identified demonstrating a relationship between breast cancer and the gut microbiome are summarized in Table 3.

**Table 3.** Bacterial species possessing the β-glucuronidase enzyme for estrogen metabolism.

Phylum	Genus	Species	Reference
Actinobacterium	Bifidobacterium	B. adolescentis	Nakamura et al.^184^
		B. breve	Nakamura et al.^184^; Stringer et al.^185^
		B. dentium	McIntosh et al.^186^
		B. longum	Nakamura et al.^184^
Bacteroidetes	Bacteroides	B. dorei	McIntosh et al.^186^; Pollet et al.^187^
		B. fragilis	Pollet et al.^187^; Stringer et al.^185^
		B. ovatus	Gloux et al.^188^; Pollet et al.^187^
		B. uniformis	Nakamura et al.^184^ Pollet et al.^187^
		B. vulgatus	Nakamura et al.^184^ Pollet et al.^187^
	Parabacteroides	P. johnsonii	Gloux et al.^188^
		P. merdae	Gloux et al.^188^; Pollet et al.^187^
Firmicutes	Clostridium	C. acetobutylicum	Girbal et al.^189^
		C. asparagiforme	McIntosh et al.^186^
		C. bartlettii	Gloux et al.^188^
		C. beijerinckii	Girbal et al.^189^
		C. clostridioforme	Nakamura et al.^184^
		C. coccoides	Luu et al.^164^
		C. difficile	Mani et al.^190^
		C. hathewayi	McIntosh et al.^186^
		C. leptum	Luu et al.^164^
		C. paraputrificum	Nakamura et al.^184^
		C. perfringens	Hartman et al.^191^; Leung et al.^192^; McIntosh et al.^186^; Nakamura^184^; Pollet et al.^187^; Shimizu et al.^193^
	Escherichia	E. coli	Leung et al.^192^; McIntosh et al.^186^; Nakamura et al.^184^; Pollet et al.^187^
	Eubacterium	E. eligens	McIntosh et al.^186^; Nakamura et al.^184^; Pollet et al.^187^
	Faecalibacterium	F. prausnitzii	Dabek et al.^194^; Gloux et al.^188^; McIntosh et al.^186^; Pollet et al.^187^
	Lactobacillus	L. gasseri	McIntosh et al.^186^; Russell et al.^195^
		L. jensenii	McIntosh et al.^186^
		L. rhamnosus	McIntosh et al.^186^
	Roseburia	R. hominis	Dabek et al.^194^
		R. intestinalis	Dabek et al.^194^; McIntosh et al.^186^
		R. inulinivorans	Gloux et al.^188^
	Ruminococcus	R. gnavus	Beaud et al.^196^; Gloux et al.^188^; McIntosh et al.^186^; Nakamura et al.^184^

Genera that stand out with potentially protective mechanisms against breast cancer include *Faecalibacterium* (phylum Firmicutes) and *Akkermanisia* (phylum *Verrucomicrobiota*). Genus *Faecalibacterium* has been identified in several breast cancer studies.^154,163,164,171^ A major commensal of the gut microbiome, *F. prausnitzii* is a producer of SCFA butyrate and shown to have anti-cancer effects in lung cancer in-vitro.^172^ Depletion of *F. prausnitzii* has been associated with Crohn’s disease, ulcerative colitis and colorectal cancer.^173,174^ In MCF-7 breast cancer cell lines, *F. prausnitzii* promoted apoptosis while inhibiting cell proliferation and invasion.^171^

Demonstrating promising probiotic and anti-inflammatory properties, *A. muciniphila* in feces has been associated with lower rates of metabolic syndrome and inflammation.^175,176^ It has been shown to increase antimicrobial peptides in the gut and thicken the mucosal layer to prevent bacterial translocation and subsequent systemic inflammation.^177^ *A. mucinophilia* is associated with gut microbial diversity in breast cancer patients^115^ and its abundance positively correlates with smaller breast tumors and node negative disease.^170^ Frugé et al. found that a higher abundance of *A. muciniphila* is associated with lower BMI and higher alpha diversity. It is also correlated with significant differences in beta diversity, higher abundance of *Prevotella* and *Lactobacillus*; reduced abundance of *Clostridium, Campylobacter* and *Helicobacter*, compared to samples with low abundance of *A. muciniphila*.^115^

Breast cancer patients have been found to have significantly elevated levels of *Clostridiales* in their feces, and high total urinary estrogen metabolite levels.^154^ While *Clostridium* species, like *Faecalibacterium*, also produce butyrate and together have been found depleted in the gut of lung cancer patients,^178^ fecal samples from breast cancer patients with higher staging were enriched in *C. coccoides* and *C. leptum*.^164^ Fuhrman et al. found that fecal levels of *Clostridia* were positively associated with metabolite-to-patient estrogen ratios, which in turn were positively associated with fecal microbial diversity.^114^ An earlier study reported a direct correlation between total urine estrogen metabolites, fecal α diversity and abundance of *Clostridia*.^162^ It may be the case that the pathogenic hormonal properties of *Clostridium* outweigh its protective effects in breast oncogenesis.

## The gut estrabolome

**Figure 2.**
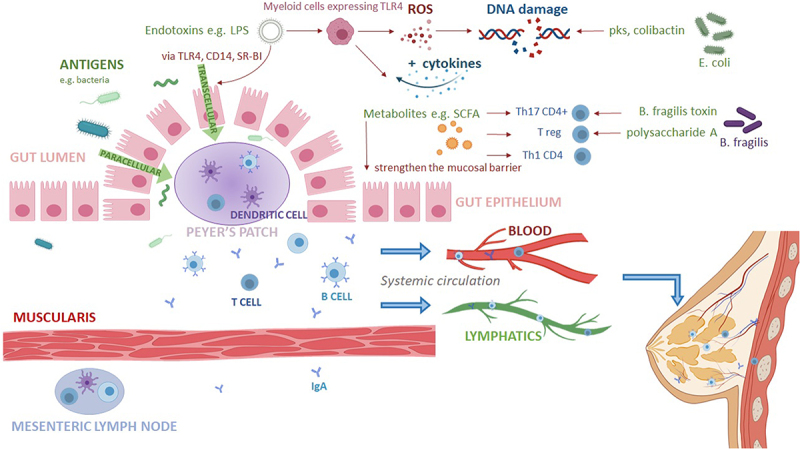
Interactions between the gut microbiome, estrabolome and systemic immune system which may contribute to or modify breast cancer risk.

The association between the gastrointestinal microbiome and breast cancer tumorigenesis is a current area of interest due to the significance of estrogen metabolizing gut bacteria, the genes of which have been referred to as the estrabolome.^179^ The estrabolome plays an important role in the enterohepatic recirculation of estrogens and systemic estrogen levels. This relationship is illustrated in Figure 2.

High estrogen states are a risk factor for breast cancer. Estrogen exposure can come from the systemic circulation or from the gut via the entero-mammary pathway. Estrogen metabolizing bacteria in the gut make up the estrabolome and are affected by various modifiable and non-modifiable risk factors. It interacts via the enterohepatic circulation to excrete or recycle estrogens back through the systemic circulation; on the other hand, gut dysbiosis can alter inflammatory immune cell activity and estrogen receptor signaling to promote neoplastic processes at the breast tumor microenvironment. Interventions to disrupt the complex interactions between the gut and breast microbiome and systemic circulation include antibiotics that alter the composition of β-glucuronidase containing bacteria in the gut and traditional breast cancer therapies.

Created with BioRender.com

Higher estrogen states are a known risk factor for ER positive breast cancer.^180^ Parent estrogens, estradiol, estrone, and their metabolites are conjugated with glucuronic acid in the liver to facilitate their excretion via urine or bile. Interestingly, higher concentrations of parent estrogens have been linked to increased post-menopausal breast cancer risk, whereas a higher metabolite to parent estrogen ratio is associated with reduced risk, particularly those metabolized via the 2-hydroxylation pathway.^181–183^

Deconjugation by β-glucuronidase containing bacteria, detailed in Table 4, facilitates the reabsorption of deconjugated estrogens back into the systemic circulation in their active forms. Notable bacteria with the β-glucuronidase enzyme to deconjugate estrogens for reabsorption include *Alistipes, Bacteroides, Bifidobacterium, Collinsella, Edwardsiella, Faecalibacterium* genera, and *Lactobacillus* and *Roseburia*.^197^ It has been hypothesized that those with a diverse gut microbiome rich in bacteria with deconjugating enzymes are at increased risk of breast cancer, due to enhanced enterohepatic reabsorption of estrogens and overall estrogen burden.^179^ Microbiome diversity and high β-glucuronidase activity in the gut have indeed been associated with higher concentrations of estrogen metabolites in urine, indicating higher systemic estrogen burden, and lower estrogen concentrations in feces.^162,198^ Microbial gut diversity is also positively correlated with a higher metabolite to parent estrogen ratio,^114^ and therefore a lower risk of post-menopausal breast cancer. Indeed, post-menopausal states appear to be associated with lower gut microbial diversity, a microbiome composition more similar to men and less gut β-glucuronidase activity, compared to pre-menopausal women.^199^ The feces of post-menopausal women with breast cancer have less microbial diversity compared to cancer-free controls, independent of estrogen.^154^ NAF from breast cancer patients showed different bacterial compositions to normal healthy controls and identified that the cancerous samples expressed higher concentrations of β-glucuronidase.^30^

**Table 4.** Summary of significant bacterial species identified demonstrating a relationship between the gut microbiome and breast cancer.

Significant Bacteria Identified (Phylum – Genus)	Study Type	Breast Cancer Study Size	Sample Type	Method of Analysis	Reference
**Firmicutes**: Clostridia	Cross-sectional	51 BC (women 19 pre, 7 postmenopausal, 25 men)	Urine Faeces	LC-MS/MS Realtime kinetics 16S rRNA amplicon pyrosequencing	Flores et al.^162^
**Bacteroidetes**: −Bacteroides **Firmicutes**: −Clostridium −Ruminococcus	Cross-sectional	60 health postmenopausal women (ages 55–69)	Urine Faeces	LC-MS/MS 16S rRNA amplicon sequencing	Fuhrman et al. ^114^
**Actinobacteria**: −Eggerthella −Actinobacteria −Bifidobacterium **Firmicutes**: −Faecalibacterium spp. prausnitzii −Blautia	Cross-sectional	32 BC patients; IDC (81%), ER+/PR+ (80%), HER2+ (15%)	Faeces	16S rRNA PCR gene sequencing	Bard et al.^163^
**Firmicutes**: −Clostridium −Faecalibactrium −Ruminococcus −Lachnospiraceae −Dorea	Case-control	48 BC postmenopausal pre-treatment 48 Control postmenopausal normal mammograms	Urine Faeces	16S rRNA gene sequencing LC-MS/MS	Goedert et al. ^154^
**Actinobacteria**:Eggerthella **Firmicutes**: −Bacteroidetes −Blautia −Clostridium −Faecalibacterium	Cross-sectional	31 early-stage BC	Faeces	16S rRNA gene sequencing DNA extraction (PSP Spin Stool DNA kit)	Luu et al.^164^
**Actinobacteria**:Actinomyces **Firmicutes**: Enterococcus **Proteobacteria**: Acinetobacter Citrobacter Erwinia Escherichia Shewanella	Population based cohort	48 postmenopausal BC (75% stage 0-I; 88% ER+) 48 Control age-matched postmenopausal with normal mammograms	Urine Faeces	16S rRNA gene amplicon sequencing high-performance LC-MS/MS radioimmunoassay	Goedert et al.^165^
**Bacteroidetes**:Bacteroides Proteobacteria −Pseudomonas −Escherichia **Firmicutes**: −Clostridium −Staphylococcus	Case-control	46 postmenopausal BC 48 Control postmenopausal 28 mice injected with 4T1 cancer cells. MCF-7, 4T1 and primary fibroblast cell lines	Human feces Mice mammary tissue	Cell proliferation assays PI assays Scratch assay ECIS, qPCR SDS-PAGE, IHC Western blot	Mikó et al.^166^
**Actinobacteria** − Actinomyces **Bacteroidetes**: − Prevotella **Firmicutes** − Enterococcus **Proteobacteria**: − Escherichia − Klebsiella − Shewanella − Erwinia	Case-control	18 premenopausal BC 44 postmenopausal BC 25 premenopausal controls 46 postmenopausal controls	Faeces	Shotgun metagenomics	Zhu et al.^119^
**Bacteroidetes** **Actinobacteria**: Bifidobacterium	Cross-sectional	124 invasive BC no chemotherapy currently (46% previous chemo)	Blood Faeces	16S rRNA gene sequencing Gas chromatography	Horigome et al.^167^
**Bacteroidetes –** **Firmicutes**:Anaeotruncus	Cross-sectional	Mice -mammary injections of PyMT-BO1 and EO771 cell lines, antibiotic cocktail (VNMA) & ampicillin Control: water-treated	Faeces	16S rRNA amplicon PCR gene sequencing Flow cytometry NMR spectroscopy	Kirkup et al.^168^
**Proteobacteria**:Escherichia	Case-control	32 female mice grafted with 4T1 BC cells (16 cadaverine treated, 16 control) **Cell lines**: MD-MBA-231, SK-BR-3, ZR-75–1, MCF-7 & 4T1 48 postmenopausal BC Control: 48 healthy women	Murine Mammary tissue Human feces	Cell proliferation assays Annexin V + PI ECIS assays, IHC RT-qPCR SDS PAGE & Western blot Wound healing assay Matrigel invasion assay TBARS assay Aldefluor assay	Kovács et al.^127^
**Proteobacteria**: − Citrobacter − Veillonella	Cross-sectional	121 BC	Faeces	Metagenomics	Yoon et al.^169^
**Verrucomicrobia**:Akkermansia	Longitudinal	32 BC patients (stage 0 – II, BMI ≥ 25;	Faeces	16S rRNA gene sequencing	Yoon et al.^115^
**Bacteroidetes**: −Verrucomicrobia **Actinobacteria** **Firmicutes**: −Faecalibacterium **Proteobacteria**	Case-control	25 BC 25 benign breast disease MCF-7 BC cell line	Faeces Blood	16S rDNA sequencing LC-MS metabolomics Chromatography-MS Cell and bacterial culture ELISA, Western blot Cell proliferation, Apoptosis & invasion assays	Ma et al.^40^
**Actinobacteria**:Collinsella **Bacteroidetes**:Bacteroides **Firmicutes**: −Clostridium −Eubacterium **Verrucomicrobia**:Akkermansia	Case control	**Cohort 1**: 76 pre-chemo– 42% HR+, 34% HER2+, 24% 45 TNBC- pre & post-chemo 54 Ctls: healthy volunteers **Cohort 2**: 27 BC (17 pre-chemo, 10 post chemo)	Faeces	Shotgun metagenomics	Terrisse et al.^170^

Annexin V + PI : Annexin V and propidium iodide (PI) labeling of cells is a technique used to identify cell death; BC: breast cancer; Chemo: chemotherapy; ECIS: Electric Cell-substrate Impedance Sensing; ER+: estrogen receptor positive; ELISA: enzyme-linked immunosorbent assay; HER2+: human epithelial growth factor receptor positive; HR+: hormone receptor positive; IDC: invasive ductal carcinoma; IHC: Immunohistochemistry; LC-MS/MS: Liquid chromatography/tandem mass spectrometry; MS: mass spectrometry; NMR: nuclear magnetic resonance spectroscopy; NSCLC: non-small cell lung carcinoma; PCR: polymerase chain reaction; PR+: progesterone receptor positive; RT-qPCR: reverse transcription polymerase chain reaction; TNBC: triple negative breast cancer.

### Entero-mammary pathway

The ‘bacterial entero-mammary pathway’, a proposed mechanism by which mononuclear immune cells (i.e. dendritic and CD18 expressing cells) migrate from the GIT to the breast, may be a carrier for gut bacteria to translocate to mammary tissue.^200^ Well established is the mucosal immune system for the migration of IgA cells between mucosal surfaces, as previously described.

The composition of the breast microbiome may be directly influenced by intestinal bacteria via this pathway and may explain how intestinal bacteria such a *Enterobacteriaceae*, a large family of gram negative bacteria often found in the gut microbiome, have been found in the breast TME.^25,26^ Oral ingestion of probiotic *Lactobacillus* strains (*L. fermentum* or *L. salivarius*) to treat mastitis led to isolation of those strains in milk samples, suggesting that there is a mechanism by which gut microorganisms are able to migrate from the gut to mammary tissue.^201,202^ Detected in human breast milk, *L. salivarius* and *L. gasseri*, were shown to cross through a Caco-2 cell monolayer (an in vitro representation of the intestinal epithelial layer) via dendritic cells.^203^ From the breastmilk of healthy lactating women, bacteria such as *Streptococcus, Lactobacillus, Enterococcus, Peptostreptococcus, Staphylococcus, Corynebacterium* and occasional *Escherichia* spp., have been isolated.^204^

The presence of enteric bacteria in milk, taken with the finding of some rDNA bands common to maternal milk, blood and feces, suggests bacterial translocation via a mononuclear-mediated mechanism.^204^ In-vivo studies have shown *L. reuteri* suppresses tumor formation when introduced to the GIT of breast cancer-prone mice with HER2 mutations, by stimulating CD4+ and CD25+ lymphocytes.^205^ 16S rRNA gene sequencing of fecal samples from women with breast cancer detected higher counts of *Clostridia* and *Blautia* sp., with increasing grade and stage.^163,164^ The interplay between enteric microorganisms and the human immune system in the promotion and inhibition of carcinogenesis may be of clinical significance. It has potential as a target for primary prevention of breast cancer, via manipulation of modifiable risk factors, such as diet and antibiotic use.

### Estrogen modification

The host’s immune cells are also influenced by the estrabolome. ER signaling has a significant influence on the activity of the innate and adaptive immune systems.^206,207^ Importantly, 17β-estradiol can cause epigenetic changes and gene transcription by complexing at DNA sites and transcription factors, especially those related to immune cell function.^208,209^ Proteins ER-α and ER-β, encoded by the ESR1 and ESR2 gene, respectively, are expressed on a variety of innate and adaptive immune cells, including CD4+ and CD8+ Tcells, B-cells, monocytes, NK cells and monocyte-derived dendritic cells, affecting immune cell growth and function.^207,210^ There is variable ER expression between different cell types with B cells found to have the highest expression of ESR1 and ESR2 RNA, together with plasmacytoid dendritic cells for the latter, while other T cells such as CD4+ and CD8+, monocytes and NK cells have positive but low or moderate expression of those genes.^210,211^ Estrogens can induce histone modifications affecting processes such as acetylation, phosphorylation and methylation,^209^ altering the gene expression of thousands of estrogen responsive elements.

Lower physiological estradiol concentrations are usually associated with the production of type 1 IFN, which regulates inflammatory pathways, and hence the production of pro-inflammatory cytokines.^212,213^ In contrast, higher physiological estrogen levels and ectopic estrogens are generally associated with inhibition of inflammatory pathways,^214–216^ Thus, in pathological states such as cancer, a TME with a higher local concentration of estrogen would suppress the production of inflammatory cytokines, dampen the immune response and promote the neoplastic process.

## Oral antibiotics

Oral antibiotics may be able to modify breast cancer risk via manipulation of the gut estrabolome. Certain antibiotics have been shown to alter the levels of specific bacteria involved in estrogen metabolism, such as *Lactobacillus* and *Bacteroides*. Changes in the gut microbiome can lead to increased levels of harmful metabolites or decreased levels of protective ones, thus impacting overall estrogen balance in the body. A large systematic review found that antibiotic exposure, particularly to penicillins, tetracyclines and nitrofurans, moderately increased breast cancer risk.^217^ Frequent antibiotic use in breast cancer survivors has been associated with an increased risk of second breast cancer events, although without clinically significant results.^218^ The net effect may be that of increased chronic inflammation and disturbed tissue metabolism.^219^ There are classes of antibiotics with anti-cancer activity, notably ciprofloxacin and gemifloxacin in TNBC and breast adenocarcinoma, respectively, *in vitro*.^220,221^ Incubation of human MDA-MB-231 breast cancer cells with increasing concentrations of ciprofloxacin demonstrated dose and time-dependent cytotoxicity while increasing concentrations of gemifloxacin progressively decreased migration and invasion of MDA-MB-231 and MDA-MB-453 cells.^220,221^

In contrast, multiple large cohort studies reported a weak relationship between long-term antibiotic use and breast cancer risk.^222–226^ Numerous studies have reported no association between antibiotic use and breast cancer risk.^227–229^ Moreover, Zackular et al.^230^ demonstrated antibiotic therapy to decrease tumor growth by reducing microbial diversity in mice.^230^

## Microbial-based therapy

The gut microbiome has proven to be malleable and dynamic. It can be hypothesized that manipulation of the gut microbiome may be a method of modifying breast cancer risk and optimizing responsiveness to systemic therapies such as chemotherapy or immunotherapy for high-risk disease.

### Chemotherapy

Fecal composition may provide crucial information about potential biomarkers for prognosis or drug resistance and may play a role in guiding chemotherapy selection. Higher levels of *Blautia obeum* in the gut may be protective against colorectal cancer risk^231^ and is associated with significantly better progression free and overall survival in hepatocellular carcinoma.^232^ However, genus *Slackia* (genus of *Actinomycetota*, in the family *Coriobacteriaceae)*, linked to early colorectal cancer and gastric cancer progression^233,234^ is associated to poorer survival outcomes. This correlation was found as well in a small study in patients with metastatic HER2 negative breast cancer undergoing capecitabine chemotherapy, reporting longer progression free survival in those with higher fecal levels of *Blautia obeum* and shorter in those with higher levels of *Slackia*.^235^ Of note, it was found that the gut microbiome composition was drastically altered by metronomic capecitabine compared to the routine dose, demonstrating significant differences in fecal microbial composition, function and reduced diversity.^235^ Species from the family *Enterobacteriaceae* are shown to inactivate doxorubicin; *Klebisella pneumoniae, Escherichia coli, Raoultella planticola*.^236^ Lower levels of *A. muciniphila* is associated with taxane-induced systemic inflammation and neuropathic pain.^237^ Abundance of *A. muciniphila* in the gut of mice injected with TNBC cell line 4T1 has been associated with responsiveness to doxorubicin, while administration of LPS, a component of gram negative bacteria, increased intestinal inflammation and reduced response to doxorubicin.^238^

Cyclophosphamide has been shown to promote inflammation in mice with breast tumors by disturbing the gut epithelium barrier, allowing the migration of *Lactobacillus johnsonii*, *Lactobacillus murinus* and *Enterococcus hirae* to lymph nodes and triggering production of Th17 and Th1 cells.^239^ Germ-free and antibiotic-treated mice demonstrated resistance to cyclophosphamide,^239^ while oral administration of *E. hirae* improved response to chemotherapy.^239,240^

### Radiotherapy

A gut microbial composition that reduces radiotherapy-related side effects may improve treatment outcomes. The gut microbiome has been revealed to have a role in radiotherapy efficacy and toxicity in the treatment of breast cancer.^117^ The degree of radiosensitivity may be determined by the immune response of the TME, with evidence that it is influenced by local microbial activity and metabolism.^241^ Macrophages can be classified as pro-inflammatory (M1) or anti-inflammatory (M2).^242^ A greater abundance of M1-polarized macrophages has been shown to increase the efficacy of radiotherapy on breast cancer cells in vitro, while M2-polarized macrophages promoted radio-resistance. Rectal microbial diversity has been shown to be predictive for responsiveness to radiotherapy in the treatment of cervical squamous cells carcinoma.^243^ Ovarian tumors with high infiltration by CD8+ T cells and M1 macrophages had a better prognosis, and a unique intratumoral microbial signature enriched in *Achromobacter, Microcella, Devosia, Ancylobacter* and *Acinetobacter*.^244^

Additionally, gut abundance of *Lactobacillus sakei, L. acidophilus, L. casei, L. reuteri* and *Bifidobacterium* spp. have been shown to be protective against radiation-induced enteritis.^245–247^

### Immunotherapy

There is growing evidence that the gut microbiota is involved in the clinical response to immune checkpoint inhibitors (ICIs). Vétizou et al.^248^ found *Bacteroides* species, particularly *B. thetaiotaomicron* and *B. fragilis*, to have a role in improving the therapeutic efficacy of anti-CTLA-4 ICIs, for the treatment of melanoma and colon cancer in mice.^248^ On the other hand, the immunosuppressive effect of *B. fragilis* has been described earlier (section Microbial Toxins). Of note, fecal *Bacteroidetes* numbers are reported to increase with increasing stage of breast cancer,^164^ making it a potential target to maximize efficacy of immunotherapy.

*Bifidobacterium* alleviates the symptoms of anti-CTLA-4 ICI-induced colitis in melanoma-afflicted mice.^249^ Gopalakrishnan et al.^250^ found the gut microbiome modulates a response to anti-PD-1 immunotherapy in melanoma patients through enhancing antigen presentation or increasing T cell recruitment to the TME. Responders exhibited a more diverse fecal microbiome with enrichment in *Ruminococcaceae, Clostridiales* and *Faecalibacterium*.^250^ The probiotics *Bifidobacterium* spp. and *A. muciniphila* were demonstrated to promote the efficacy of anti-PD-1 therapy against epithelial tumors.^251,252^

In more recent years, the utility of ICIs in treatment of breast cancer is expanding, demonstrating improved clinical outcomes. A systemic review indicated that specific gut signatures may be predictive of a good response to immunotherapy. For example, patients with a baseline gut abundance of *Bacteroides fragilis*, *Streptococcus* and *F. prausnitzii* had better clinical responses to hormone therapy and pembrolizumab.^253^ Characterizing the immune profile of the TME will expand the potential of immunotherapy in breast cancer treatment.

### Targeted therapy

*Clostridiales* appears to be significant in the treatment of HER2 positive breast cancers, noting a greater abundance of *Clostridiales* and less *Bacteroidales* in the feces of patients who achieved pathological complete response with neoadjuvant trastuzumab.^254^ The proposed mechanism is that commensal gut bacteria may enhance the response to monoclonal antibody therapy via greater recruitment of immune cells to the TME. Trastuzumab was also more efficacious in the presence of *L. lactis* or *L. paracasei*.^254^

### Hormone therapy

The effect of the gut microbiome on hormone therapy efficacy has been observed in hormone dependent cancers. In prostate cancer, dysbiosis involving androgen-producing microbes are able to degrade androgen deprivation therapy.^255,256^ In ER positive breast cancers, differences were noted in the gut microbial composition of good responders to adjuvant aromatase inhibitor therapy compared to poor responders, with poor responders demonstrating higher gut microbiome diversity and elevated levels of *Veillonella*.^257^ *Veillonella* (phylum Firmicutes) in the gut has been associated with poor prognosis in hematology patients treated with anti-CD19 chimeric antigen receptor (CAR) T cell therapy, and in tumor tissue associated with poor prognosis in lung cancer.^258,259^

A study of the fecal microbiome of HR positive breast cancer patients reported several bacteria that may be clinically relevant. Fecal enrichment with several members of the family *Clostridiaceae* was associated with node positive disease and resistance to ICIs in breast cancer patients. On the other hand, *Eubacterium rectale, Methanobrevibacter smithii, Coprococcus comes, Coprococcus catus* and *Collensella aerofaciens* were associated with better prognosis; *Eubacterium rectale, Eubacterium eligens, Eubacterium ventriosum* and *Collinsella aerofaciens* were shown to inhibit the growth of breast cancer cells in vitro.^170^

### Faecal microbial transplants

Specialized diets, bacterial probiotic “crapsules” or FMTs are hypothesized to nurture a ‘favourable’ gut microbiome, which may have a role in breast cancer treatment or even primary prevention.^260,261^

Implantation of a favorable microbiome may be the mechanism by which FMTs are able to enhance the immune response. Giving FMTs from epithelial cancer patients who responded well to ICIs to germ free or antibiotic-treated mice facilitated anti-PD-L1 efficacy and increased relative abundance of *A. muciniphila*.^251^ A similar study was replicated in renal cell carcinoma patients, whereby feces from good responders to immunotherapy were abundant in *A. muciniphila* and *B. salyersiae*, and FMTs given to antibiotic-treated mice induced significant responses to immunotherapy compared to FMTs from non-responders (100% compared to 40%, respectively).^262^ Melanoma studies have linked an abundance of *A. muciniphila, Bifidobacterium longum* and *Enterococcus faecium* with good clinical response to anti-PD1 therapy.^250,263^ Both oral supplementation of bacteria and FMTs from human responders given to antibiotic-treated or germ-free mice improved response to anti-PD-L1 therapy.^250,263^

FMTs from breast cancer patients to mice inoculated with AT3 breast cancer cells led to poor response to chemotherapy, contrasting to outcomes in mice given FMTs sourced from healthy volunteers.^170^ Furthermore, cohabitating both groups of mice improved the effects of chemotherapy on mice treated with FMTs from breast cancer patients. The FMTs from healthy volunteers had more abundance of *Eubacterium rectale*, *Methanobrevibacter smithii*, *Coprococcus comes* and *C. catus*. This study included patients with a mix of breast cancer subtypes, and subgroup analysis based on HR and HER2 status was not done. This could be important to flesh out in future studies, given the effect the gut microbiome may have on systemic estrogens and immune cells.

The efficacy of trastuzumab in mice with HER2 positive breast tumors was impaired by the administration of antibiotics, an effect that was reversed by FMTs from trastuzumab responsive mice to non-responsive mice.^254^ Treatment responsive mice had higher serum levels of IL-12, a Th1 cytokine which induces effector T and NK cells, after trastuzumab exposure. The immune-mediated effect of antibiotics on trastuzumab efficacy was supported by the significant decrease in intra-tumoral CD4+ T cells in antibiotic-treated mice. Deceased levels of *Lachnospiraceae*, *Actinobacteria*, *Turicibacteraceae* and *Bacteroidetes* were found in antibiotic-treated mice, a result which was also found in human patients with HER2 positive breast cancer who were poor responders of trastuzumab in the neoadjuvant setting. While this was a small study of only 24 human patients, the significance of these findings may be of clinical relevance, particularly the importance of *Lachnospiraceae* and *Bacteroidetes* which are also less abundant in poor responders of anti-PD1 melanoma treatment.^250^

### Extracellular vesicles

Extracellular vesicles (EVs) are formed when cells break off some of their intracellular material proteins (lipids, genetic material or sugars) and package it in its own cellular membrane. Once independent from the parent-bacteria, EVs can communicate inter-cellularly by macropinocytosis, endocytosis or membrane fusion.^264^ EVs can transport bacterial intracellular contents around the body in body fluids, becoming a vehicle for communication between different cells and organs.^265^

Bacteria produce EVs, both in pathological and physiological conditions.^265^ In the case of pathogenic EVs, as the EV membrane is taken from its parent-bacteria, they also present pathogen-associated molecular patterns and hence can be detected by the body’s immune cells via pattern recognition receptors. This can result in an inflammatory response by induction of the innate and adaptive immune responses.^266–270^

Intestinal bacterial EVs are able to cross the gut epithelium, interact with local immune cells, as well as disseminate systemically via blood, lymph or the hepatobiliary system,^271,272^ with or without a compromised gut epithelium.^273^ Patients with chemotherapy-induced colonic mucositis, HIV or inflammatory bowel disease were found to have higher concentrations of serum EVs,^274^ likely due to increased gut permeability from a compromised intestinal barrier.^275^ A study of diabetic patients reported lower serum levels of EVs derived from *A. muciniphila*, a protective commensal of the gut epithelium.^276^ Meanwhile, feeding mice EVs derived from *A. muciniphila* was found to decrease gut permeability.^276^

A review of studies on the utility of EVs as a biomarker for breast cancer found that while serum levels of EVs were consistently elevated, it was not associated with stage or subtype.^277^ EVs from metastatic breast cancer cells *in vitro* increased cell migration and invasion.^278,279^ Their contents, which have been found to contain oncogenic proteins including Epidermal Growth Factor Receptor and miRNAs, have been suggested as an alternative and more specific biomarker for breast cancer.^280–282^ Elevated levels of the HER2 protein have been found in EVs produced by breast cancer cells lines expressing the HER2 encoding gene ERBB2.^283^ Additionally, breast cancer cells resistant to chemotherapy have been demonstrated to be able to transfer their treatment resistance to sensitive cancer cells via EVs.^284–286^ Hence, peripheral EVs could be a novel strategy to both monitor and promote treatment efficacy.

In recent years, there has been growing interest in the use of EVs as a therapeutic vehicle, mainly in the context of vaccination. An avenue of microbial-based therapy being explored in the field of oncology is FMT, aiming to colonize the gut with favorable bacteria.^287^ Administration of bacterial EVs could potentially be a better and safer alternative, as they have greater stability across temperatures and do not replicate in vivo while still sharing the same immunogenic membrane and contents derived from its parent-bacteria.^288^ Notably, the binding of *B. fragilis* to TLR2 promotes Treg differentiation^105^ and administration of *B. fragilis*-derived EVs in mice mimic this same effect.^289^ Administration of modified *E. coli* EVs has been shown to stimulate production of CXCL10 and IFN-γ, thus inducing an anti-tumoral immune response.^290^ There is *in vitro* evidence of the benefit of probiotic bacterial EVs in treating hepatocellular cancer. EVs from *L. rhamnosus* was shown to have a cytotoxic effect on the HepG2 liver cancer cell line.^291^ Incubation of MCF-7 and BT474 breast cancer cell lines with β-galactosidase producing *K. pneumoniae* and *S. aureus*-derived EVs enhanced tamoxifen efficacy, via downregulation of cyclin E2, p-ERK and p21.^292,293^ Similarly, in TBC cell lines MDA-MB-231 and 4T1, bacterial EVs from *E. coli* have been demonstrated to inhibit tumor cell growth, invasion and migration, decrease Treg cells and promote apoptosis both in *in vivo* murine models.^294–296^ Hence, bacterial EVs may have utility as an adjunct to immunotherapeutic and chemotherapy agents.

## Conclusion

There is a highly complex dynamic interaction between the gut microbiome, the immune system, estrabolome and the breast TME. Future studies should aim to establish stronger evidence through mechanistic studies to extend our understanding beyond that of the current associations.

The evidence in the literature suggests that both the breast and gut microbiota play a significant role in modulating breast tissue homeostasis. While many genera and species have been named as potentially significant, some of which have associations with other cancers or other disease states, causative links to breast cancer are still largely unknown or poorly defined. There is potential for the application of certain microbial signatures as biomarkers of disease. Species highlighted in this review that may be of particular interest for future studies are *M. radiotolerans* and *S. yanoikuyae* in breast tissue, and *A. muciniphila* in the gut. The microbiome can affect drug responsiveness to systemic chemotherapy, radiotherapy and hormonal therapies as well as modulate the immune system. Elucidating the mechanisms could have massive clinical implications on the future prevention, screening, treatment, and prognostication of breast cancer.
